# Exploring the relationship between spiritual well-being and death anxiety in patients with gynecological cancer: a cross-section study

**DOI:** 10.1186/s12904-021-00778-3

**Published:** 2021-06-01

**Authors:** Yue Feng, Xingcan Liu, Tangwei Lin, Biru Luo, Qianqian Mou, Jianhua Ren, Jing Chen

**Affiliations:** 1 Department of Nursing, West China Second University Hospital, Sichuan University, Chengdu, China; 2 Key Laboratory of Birth Defects and Related Diseases of Women and Children, Ministry of Education, Sichuan University, No.20, Section 3, South Renmin Road, Chengdu, China; 3 West China School of Nursing, Sichuan University, Chengdu, China; 4 Department of Gynecology Nursing, West China Second University Hospital, Sichuan University, No.20, Section 3, South Renmin Road, Chengdu, West China China; 5 Good Clinical Practice Center, West China Hospital, Sichuan University, Chengdu, China

**Keywords:** Spiritual health, Spiritual well-being, Spirituality, EORTC

## Abstract

**Background:**

In recent years, spiritual well-being has gradually gained the attention of health care providers in China, especially those in oncology departments, who have recognized the importance of improving spiritual well-being in cancer patients. Since most of the current research on spiritual well-being has been carried out in areas with religious beliefs, this study was conducted in the context of no development of formal religion. The purpose of this study was to explore the relationship between death anxiety and spiritual well-being and the related factors of spiritual well-being among gynecological cancer patients.

**Methods:**

This cross-section study was conducted among 586 gynecological cancer patients. The European Organization for Research and Treatment for Cancer Quality of Life Questionnaire-spiritual well-being32 (EORTC QLQ-SWB32) and Templer's Death Anxiety Scale (T-DAS) were used to measure spiritual well-being and death anxiety. The Multiple Linear Regression Model was used to determine the relationship between spiritual well-being and death anxiety.

**Results:**

For all participants, the highest QLQ-SWB32 centesimal score was 75.13 on the Relationship with Other scale, and the lowest was 60.33 on the Relationship with Someone or Something Greater Scale. The mean Death Anxiety score was 5.31 (SD 3.18). We found that Relationship with Someone or Something Greater was the only scale not associated with death anxiety. Overall, patients with lower death anxiety have a higher level of spiritual well-being. Besides, a high Relationship with Other score was associated with living with a partner (B = 2.471, *P* < 0.001) and married (B = -6.475, *P* = 0.001). Patients with higher Global-SWB were retired (B = 0.387, *P* = 0.019).

**Conclusions:**

Our study found that the spiritual well-being of patients with gynecological cancer in China was no worse than in other countries with religious beliefs and patients with lower death anxiety have a higher level of spiritual well-being. Clinical staff should pay attention to the spiritual health of cancer patients, and spiritual care should be regarded as an essential element in cancer care.

## Background

Gynecological cancers are severe and potentially life-threatening illnesses, and the most common types are cervical, uterine, and ovarian cancers[1]. There are nearly 247,047 new cases of gynecological cancers (including ovary, uterus, and cervix) and 96,579 deaths in 2020 in China and with an increasing trend[2]. Although the survival rate of cancer has been improved with advancements in medical sciences, being diagnosed with cancer is still a distressing experience for these patients. As a dangerous stressor, cancer can make patients feel the threat of death and produce negative emotional experiences related to death[3].

According to the terror management theory (TMT)[4], conditions that remind people of death make them quickly experience anxiety, which comes from a reduced sense of safety and intense fear[5]. It is reported that death anxiety can bring adverse consequences, such as being associated with depression, aggravating distress specifically in cancer patients[6], and can compromise the quality of life[7]. However, the theory asserts that people can alleviate this anxiety feeling that their lives are full of meaning and purpose[8]. Extensive studies have proved this assertion that a stronger feeling of life meaning correlates with a minor degree of death anxiety[8, 9].

Spiritual well-being (SWB), another factor contributing to the quality of life of cancer patients, has garnered increasing attention[10]. Spirituality refers to the way individuals seek and express meaning and purpose in life and how they experience their connectedness to the moment, to self, to others, to nature, and the significant or sacred[11]. A previous study integrated reviews of the concept of spirituality[12] identified its themes as existential reality (experiences, meaning and purpose in life, hope), connectedness/relationship (with self, others, nature, and higher being), transcendence (level of awareness, a rising above of going beyond the limits of material existence), power/force/energy (creative energy, motivation, a striving for inspiration). We found from the concept of spirituality that it contains many dimensions, of which the dimension of meaning and purpose in life is negatively related to death anxiety[8, 9]. So, we propose a hypothesis that people with high spiritual well-being have low death anxiety.

According to Fisher[13], spiritual well-being can be understood as a fundamental dimension of people's overall health and well-being, and an indicator of the spiritual quality of life, permeating and integrating all the other dimensions of health (physical, psychological, and social). Literature has indicated that spiritual well-being can increase the resistance to mental health crises in cancer patients in the process of disease diagnosis and treatment[14]. And it is also reported to impact reducing anxiety and depression, slowing down the development of cancer, and improving the quality of life[15, 16].

However, although studies have shown that spiritual care from medical staff is associated with improved quality of life[17], addressing spiritual issues is not a priority for them, which may be due to their confusion about spirituality and religious beliefs[18]. Spirituality in the Chinese context has always been concerned about Chinese cultural values[19]. For example, in Chinese culture, the word "spirit" can refer to *Qi* based on Taoism, that is, the "energy" full of heaven, earth, the universe, and nature[20]. There are also several words corresponding to the same concept of the western definition of spirituality in Chinese, such as *da-wo* (balancing external environment), *xiao-wo* (internal environment), and *tian-ren-he-yi* (the universe, nature, body, and soul of self-communion as a whole integer and holistic transcendence)[21]. Besides, China's spirituality also includes China's religious nature. Confucianism, Taoism, and Buddhism profoundly influence the development of Chinese religion and even secular culture. Therefore, there is no clear boundary between Chinese Religions (especially Buddhism and Taoism) and local famous folk religious customs[22]. A survey of 7021 Chinese found that only 15% of them are firm atheists and have never participated in any religious activities[23]. However, some studies have found that most Chinese people claim that they have no religious belief[24, 25]. That is to say; many Chinese people declare themselves to be nonbelievers but show religiously-like beliefs and behaviors[22]. Also, there is much debate about whether Confucianism, Taoism, and Buddhism are regarded as religions[21, 26]. These suggest that there is ambiguity in the Chinese understanding of whether spirituality is a cultural or a religious connotation[19]. It is this unique cultural context that provides the Chinese with a spiritual function similar to religion. It has mapped a road for the Chinese, leading to spiritual development and nurturing and enhancing their spiritual maturity[24].

In recent years, spiritual well-being has gradually gained the attention of health care providers in China, especially those in oncology departments, who have recognized the importance of improving spiritual well-being in cancer patients. Since most of the current research on spiritual well-being has been carried out in areas with religious beliefs, this study was conducted in the context of no development of formal religion.

Therefore, the purpose of this study was to investigate the relationship between spiritual well-being and death anxiety in Chinese gynecological cancer patients and to analyze the related factors affecting spiritual well-being to help us better understand the connotation of spiritual well-being and provide evidence for improving the spiritual well-being of patients, reducing death anxiety in cancer patients who are spiritual but not religious.

## Methods

### Participants and Procedures

This was a cross-section study. We recruited participants from February 2020 to July 2020 in West China Second University Hospital of Sichuan University, a women's and children's medical center in western China. Patients were enrolled if they 1) had been diagnosed with gynecological cancer, including ovary cancer, cervical cancer, endometrial cancer, trophoblastic tumor (malignant), fallopian tube cancer, etc.; 2) were aged 18 years or more, and 3) were able to understand and answer relevant questions. Patients were excluded if they 1) refused to participate in this survey and 2) had cognitive impairment or other severe organic diseases.

### Sample Size

It was recommended that a case-to-independent variable ratio of 40 to 1 is reasonable using stepwise regression techniques to assess the relationship between one dependent variable and several independent variables[27]. So, we aimed to recruit a minimum of 450 patients with gynecological cancer: 40 participants for each independent variable (40*10 = 400), plus an additional 50 to allow for participant dropout or excessive missing data.

### Measures

#### Spiritual Well-being

The European Organization for Research and Treatment for Cancer Quality of Life Questionnaire-spiritual well-being32 (EORTC QLQ-SWB32) was a measure of spiritual well-being. It was developed by the European Organization for Research and Treatment of Cancer (EORTC) based on relevant literature, expert opinion, and interviews with palliative cancer patients[28] and validated in a multilingual and cultural context, involving a total of 18 countries[29]. The QLQ-SWB32 contains 32 items, with 22 of them composing four scoring scales: Existential (e.g., "My life is fulfilling") (6 items); Relationship with Self (e.g., "I feel lonely") (5 items); Relationship with Others (e.g., "I'm able to trust others") (6 items); Relationship with Someone or Something Greater (e.g., "I believe in life after death") (5 items). These items are scored from 1 (not at all) to 4 (very much). The other ten items form a Global-SWB scale, two "skip" items that screen for current or past belief in God or someone or something greater, three of them that are only answered by those who answered "yes" to the screening items, and four non-scoring clinically relevant items[30]. The final item is used to reflect the overall SWB, which asks patients to answer from 1 (very poor) to 7 (excellent), and with an additional choice of 0 (cannot answer or be unknown). The QLQ-SWB32 is a profile-based, not preference-based, measure, so these sub-scales are each scored separately; summing scales is not appropriate[29]. The QLQ-SWB32 has been translated into the Chinese version and has been proved to have good reliability and validity[31].

#### Death anxiety

Templer's Death Anxiety Scale (T-DAS) was developed by Donald Templer in 1970 and is one of the most common tests used to measure death anxiety[32]. It consists of 15 true/false statements (e.g., "I'm very much afraid to die"), six items are reverse scored[5]. Higher total scores indicate more significant death anxiety. The scale was reported to have a reliability of 0.83 and reasonable internal consistency[33]. After a cross-cultural adaptation in 2010, the T-DAS was translated into Chinese[34]. In this study, we used the Chinese version of the T-DAS.

#### Demographic and medical information

We collected participants' basic information and clinical data such as age, marital status, working status, living with a partner, and clinical data such as diagnosis were collected from the medical record or self-report of participants. We also asked whether patients had religious beliefs or were actively engaged in religious practices.

### Statistical analysis

We performed statistical analysis using SPSS version 26.0 (IBM, Armonk, NY, USA). In our study, the patient's demographic characteristics were presented as frequency and percentages. The scores of the two measurement tools were tested for normality and showed as mean, standard deviation, median, and the interquartile range. We assessed the reliability for both scales using Cronbach's alpha coefficients to estimate whether these scales are indeed appropriate measures for our sample. Considering the differences in the possible range of each sub-scale score of QLQ-SWB32, we converted the total scores of each sub-scale into centesimal scores ([mean score/possible highest score] *100) to compare each with others. Besides death anxiety, the patient's sociodemographic characteristics were also considered factors that could influence spiritual well-being. Associations between death anxiety, spiritual well-being, and the patient's sociodemographic characteristics were first analyzed. The correlation between each sub-scale of spiritual well-being and continuous variables was calculated using the Spearman correlation coefficient. Since the scores of the QLQ SWB32 didn’t conform to a normal distribution, the relationships between spiritual well-being and classification variables were tested using Nonparametric Test. Mann–Whitney U tests were used for comparison between two groups; Kruskal- Wallis H tests were used for more than two groups. A Bonferroni correction for multiple comparisons was used. The Multiple Linear Regression analysis was run using the multiple regression stepwise procedures to find factors affecting SWB. Only variables with *P* < 0.05 in the correlation analysis were included. All statistical tests were two-tailed, and *P* < 0.05 was considered statistically significant.

### Ethical considerations

The study protocol has been approved by the hospital's ethics committee (code number: 2019–13). Our study was performed in accordance with the Declaration of Helsinki and following relevant guidelines and regulations. Each participant was fully informed of the study risks and benefits and provided written consent.

## Results

### Patient Demographics and Clinical Characteristics

Overall, a convenience sample of 586 patients with gynecological cancer participated in this study. All of them were women, with a mean age of 49.38 ± 11.06, ranging from 18 to 74. The participants we included had more than five cancer types, with a majority of Ovarian cancer (50.3%), most were married (87.4%) and live with a partner (72.5%). 90.4% of the participants declared that they had no religious beliefs, 45 (7.7%) were Buddhist, 3 (0.5%) were Christian, and 8 (1.4%) did not state their specific religious beliefs (Table 1).

**Table 1:** Demographic and Clinical Characteristics of Participants (*N* = 586)

Variables	n	%
Age		
35 y and below	82	14.0
36–45 y	90	15.3
46–55 y	240	41.0
More than 55 y	174	29.7
Marriage status		
Married	512	87.4
Others	74	12.6
Working status		
Retired	162	27.6
Laid-off	272	46.4
Employed	152	26.0
Education Background		
junior and below	305	52.0
senior middle school	123	21.0
college and above	158	27.0
Primary cancer		
Ovarian	295	50.3
Cervical	128	21.8
Endometrial	70	11.9
Fallopian Tube	38	6.5
Stage of Cancer		
I	111	18.9
II	85	14.5
III	255	43.6
IV	135	23.0
Time after Diagnose		
1 y or below	457	78.0
1–3 y	76	13.0
3–5 y	30	5.1
more than 5 y	23	3.9
Religious Belief		
Yes	56	9.6
No	530	90.4
Living with a partner		
Yes	425	72.5
No	161	27.5

### Measurement Level

Table 2 presents the means, standard deviation, median, the interquartile range for each measurement scale. For all participants, the highest QLQ-SWB32 centesimal score was 75.13 on the Relationship with Other scale, and the lowest was 60.33 on the Relationship with Someone or Something Greater scale. The Cronbach's alpha of the QLQ-SWB32 in our sample is 0.751. The mean Death Anxiety score was 5.31 ± 3.18 (Table 2), and the Cronbach's alpha for Death Anxiety in our sample is 0.756.

**Table 2:** Descriptive statistics for study scales and sub-scales (*N* = 586)

Scale	Numbers of items	Response Range	Mean	SD	Centesimal score	Median	IQR
1.QLQ-SWB32							
Existential	6	1–4	17.73	3.33	73.88	18.00	15.00–20.00
RwS	5	1–4	14.78	2.62	73.90	15.00	13.00–16.00
RwO	6	1–4	18.03	3.30	75.13	18.00	16.00–20.00
RwSG	5	1–4	12.07	2.43	60.33	12.00	10.00–14.00
Global-SWB^a^	10	0–7	5.13	1.38	73.35	5.00	4.00–6.00
2.Death Anxiety	15	0–1	5.31	3.18	-	5.00	3.00–8.00

Abbreviations: *SD* standard deviation; *IQR* interquartile range; *RwS* Relationship with self; *RwO* Relationship with others; *RwSG* Relationship with Someone/something Greater; *SWB* spiritual well-being

^a^Global SWB was only for those who responded to this question (*n* = 557)

### Related Factors of Spiritual Well-being

We analyzed the associations between each sub-scale of spiritual well-being and death anxiety, patient demographic characteristics (Table 3). The Existential scale and the Relationship with Self scale were possibly related to death anxiety. Besides being linked to death anxiety, the Relationship with Others scale may also relate to being married and living with a partner. The Global SWB was possibly associated with death anxiety, age, working status, and living with a partner (*P* < 0.05 for all).

**Table 3:** Correlation Between SWB and Death Anxiety, Participant Characteristics^a^

		Death Anxiety	Existential	RwS	RwO	RwSG^b^	Global SWB^c^
Death Anxiety	Continuous	-	-0.257**	-0.389**	-0.177**	-0.028	-0.354**
Participant Characteristics							
Age	Continuous	-0.135**	-0.029	-0.040	0.080	0.130	0.142**
Marriage Status		-1.349	-0.130	-0.002	-2.215*	-0.942	-0.811
	Married	5.24 (3.18)	73.93 (13.94)	73.96 (13.36)	75.63 (13.76)	60.48 (11.81)	73.49 (19.97)
	Others	5.77 (3.18)	73.59 (13.52)	73.45 (11.19)	71.73 (13.20)	59.20 (14.63)	72.42 (17.83)
Working Status		2.110	1.346	0.255	5.135	1.479	16.690**^c^
	Retired	5.11 (3.06)	74.28 (13.94)	73.40 (12.67)	76.34 (12.96)	62.02 (11.93)	78.39 (18.99)
	Laid-off	5.27 (3.36)	73.19 (13.93)	74.45 (14.55)	73.88 (14.09)	59.37 (12.45)	71.87 (20.36)
	Employed	5.59 (2.99)	74.73 (13.74)	73.45 (10.63)	76.10 (13.80)	60.39 (11.90)	70.65 (18.36)
Education Background		8.663*^c^	0.423	0.785	3.007	0.185	0.604
	junior and below	4.95 (3.22)	73.59 (13.88)	74.26 (14.34)	74.34 (14.27)	57.75 (11.97)	70.30 (15.20)
	senior middle school	5.45 (2.89)	73.88 (13.12)	72.85 (12.03)	76.49 (11.93)	57.89 (12.23)	69.45 (25.43)
	college and above	5.88 (3.25)	74.47 (14.51)	74.02 (11.31)	75.61 (13.98)	58.45 (12.18)	69.24 (23.62)
Primary Cancer		0.500	5.994	8.070	2.620	3.217	0.241
	Ovarian	5.31 (3.28)	73.74 (14.69)	73.53 (14.04)	75.83 (13.82)	59.82 (12.08)	72.56 (20.85)
	Cervical	5.37 (3.15)	73.96 (13.30)	74.10 (11.56)	74.02 (14.38)	59.07 (13.29)	75.03 (17.86)
	Endometrial	5.11 (3.02)	76.85 (11.34)	73.29 (13.32)	76.25 (12.70)	64.25 (12.06)	75.91 (19.14)
	Fallopian Tube	5.39 (3.41)	73.03 (14.72)	72.11 (11.95)	74.34 (14.58)	62.14 (10.69)	75.29 (20.38)
Stage of Cancer		4.496	1.748	5.411	0.862	6.640	4.729
	I	5.59 (3.12)	73.57 (14.59)	75.63 (11.16)	74.21 (13.73)	55.63 (11.85)	70.53 (22.98)
	II	5.79 (3.22)	72.06 (12.86)	71.29 (12.56)	74.95 (13.46)	58.65 (12.50)	67.73 (25.34)
	III	5.11 (3.34)	74.58 (14.15)	74.06 (14.18)	75.41 (13.63)	58.90 (12.00)	71.09 (26.31)
	IV	5.14 (2.89)	74.01 (13.40)	73.81 (12.66)	75.49 (14.21)	57.70 (11.94)	68.25 (23.00)
Time after Diagnose		1.678	1.324	1.334	2.220	4.538	0.374
	1 y or below	5.29 (3.22)	73.98 (13.68)	74.30 (13.71)	4.68 (13.74)	7.58 (12.34)	69.71 (25.19)
	1–3 y	5.68 (3.32)	74.23 (15.19)	72.96 (10.84)	76.32 (14.21)	60.26 (11.07)2	68.42 (26.01)
	3–5 y	4.60 (2.54)	73.19 (14.71)	72.00 (10.22)	76.25 (13.93)	56.50 (11.61)	72.38 (19.83)
	more than 5 y	5.30 (2.74)	71.92 (12.89)	71.52 (10.60)	78.80 (11.64)	60.00 (9.53)	73.91 (18.60)
Religious Belief		-0.533	-0.438	-0.971	-0.380	-1.688	-0.114
	Yes	5.54 (3.19)	73.07 (13.99)	75.36 (12.93)	74.55 (14.29)	63.30 (13.50)	72.49 (21.47)
	No	5.28 (3.18)	73.98 (13.88)	73.75 (13.12)	75.20 (13.69)	59.41 (11.58)	73.45 (19.52)
Living with a Partner		-0.848	-1.893	-0.933	-2.752*	-0.173	-2.970*
	Yes	5.22 (3.10)	74.59 (13.92)	74.29 (13.78)	76.08 (13.43)	60.20 (11.64)	74.90 (19.25)
	No	5.53 (3.39)	72.05 (13.64)	72.86 (11.09)	72.64 (14.25)	60.69 (13.49)	69.30 (20.32)

Abbreviations: *RwS* Relationship with self; *RwO* Relationship with others; *RwSG* Relationship with Someone/something Greater; *SWB* spiritual well-being

^a^Data were presented as Mean (SD); Mann–Whitney U tests were used for two independent samples; Kruskal- Wallis H tests were used for more than two independent samples; The correlation between continuous data was calculated using the Spearman correlation coefficient

^b^Global SWB was only for those who responded to this question (*n* = 557)

^c^Multiple comparisons were corrected for significance using the Bonferroni method. The differences between the distribution of Global SWB were statistically significant of *retired* and *laid off* (adjusted *P* = 0.002), *retired* and *employed* (adjusted *P* = 0.005). The difference in the distribution of Death Anxiety between *junior and below* and *college and above* (adjusted *P* = 0.014) was statistically significant

^*^indicates *P* < *0.05,* **indicates *P* < 0.001

The Multiple Linear Regression analysis (Table 4) was run using the multiple regression stepwise procedure; variables with *P* < 0.05 in Table 3 were included. We found that death anxiety was a significant predictor for the Existential scale and the Relationship with Self scale (B = -1.180, *P* < 0.001, and B = -1.460, *P* < 0.001). High Relationship with Other score was associated with lower death anxiety (B = -0735, *P* < 0.001), living with a partner (B = 2.471, *P* < 0.001), and married (B = -6.475, *P* = 0.001). Global-SWB was negatively associated with death anxiety (B = -0.161, *P* < 0.001). Besides, patients with higher Global-SWB were retired (B = 0.387, *P* = 0.019). The independent variables in this multiple regression analysis explained 7.3% of the variance in the Existential scores, 12.6% in the Relationship with Self scores, 5.8% in Relationship with Others, and 10.6% in Global-SWB.

**Table 4:** Stepwise Regression Analysis of Death Anxiety and Patient Characteristics with QLQ-SWB 32 Scales

Dependent variables	Model	Independent Variables	B^a^	95%CI^a^	Sig	R	R^2^	Adjusted R^2^
Existential	1	Death Anxiety	-1.180	-1.522, -0.839	< 0.001	0.271	0.073	0.072
RwS	1	Death Anxiety	-1.460	-1.773, -1.147	< 0.001	0.355	0.126	0.124
RwO	1	Death Anxiety	-0.738	-1.084, -0.392	< 0.001	0.171	0.029	0.028
	2	Death Anxiety	-0.735	-1.079, -0.391	< 0.001	0.195	0.038	0.035
		Living with a Partner	1.428	0.218, 2.638	0.021			
	3	Death Anxiety	-0.695	-1.037, -0.353	< 0.001	0.241	0.058	0.053
		Living with a Partner	2.471	1.137, 3.804	< 0.001			
		Marriage Status	-6.475	-10.116, -2.835	0.001			
Global-SWB^b^	1	Death Anxiety	-0.164	-0.206, -0.122	< 0.001	0.301	0.091	0.089
	2^c^	Death Anxiety	-0.161	-0.203, -0.119	< 0.001	0.325	0.106	0.100
		Living with a Partner	0.247	-0.055, 0.549	0.109			
		Retired	0.387	0.063, 0.710	0.019			
		Employed	0.024	-0.304, 0.353	0.884			
Death Anxiety	1	Age	-0.044	-0.067, -0.021	< 0.001	0.153	0.023	0.022

Abbreviations: *RwS* Relationship with self; *RwO* Relationship with others; *SWB* spiritual well-being

Categorical variables were coded as living with a partner: yes = 1, no = 0; marriage status: married = 0, others = 1; The working status was converted to 3 dummy variables

^a^Unstandardized Coefficients Beta with 95% Confidence Interval for B

^b^Global SWB was only for those who responded to this question (*n* = 557)

^c^Forced entry method was used for multiple linear regression analysis in model 2 of Global-SWB for it contained dummy variables

## Discussion

It was demonstrated that our participants scored better on Existential and Relationship with Self but lowered on Relationship with Someone or Something Greater compared with the scores of females with incurable cancer in a previous study from 14 countries, including China using QLQ-SWB32[30]. And the scores of Relationship with Someone or Something Greater in this study were also the lowest (60.33) compared with other sub-scales. It is similar to the results of a study of spiritual well-being in Chinese female patients with chronic diseases, who scored lower on the response to religious beliefs[35]. It might be related that 90% of the participants in this study had no religious beliefs. According to the literature, 95% of Americans believed in God or something greater, while only 4% of the Chinese believed the same[36]. So, Chinese people are less religion-oriented than those in Western countries. And we found that participants having religious faith scored higher on the Relationship with Someone or Something Greater scale. It was consistent with a previous study among Chinese patients with gynecological cancer that religious beliefs correlate with spiritual well-being [25], which may be related to the belief that God or a greater power will give strength and help inner peace. Although the majority of people in China are not religious, they are also different from those who do not adhere to any religion in other countries because Chinese people are influenced by some Chinese traditional culture, including Confucianism, Taoism, and Buddhism. There is no clear boundary between these religions and local famous folk religious customs[22]. Many Chinese people claim themselves nonbelievers, in this kind of culture but show religiously-like beliefs and behaviors[22]. For example, Buddhism emphasizes Karma, a kind of state of a relationship, responding to and reflecting the causes and results of destiny. Many Chinese patients used Karma to explain why they are hospitalized (everyone has his fate, and inevitable events in life are predetermined by a higher being when a person is born)[21]. Taoists worship their ancestors as well as the local gods to solve their life problems. But many Chinese will also pray to responsible gods based on Taoism, according to their wishes, such as the God in charge of pregnancy, or the God in charge of wealth, health, passing exams, environment, and even kitchen[37]. This particular cultural background provides the Chinese with a spiritual function similar to religion[24]. In this kind of culture, the Chinese try to find the true meaning of life, seek self-worth in the world, and even explore the essence of human beings[38]. It may explain our patients' higher scores on the sub-scales of Existential and Relationship with Self than other studies compared to other studies conducted in western countries.

In our study, the mean score of death anxiety was 5.31. It was similar to a study in Chinese cancer patients using the same instrument (5.13)[39] but higher than another study using Templer's Death Anxiety Scale in elderly Chinese female people (3.69)[40]. This may relate to that cancer can make patients feel a strong threat of death and produce anxiety about death[3]. The Relationship with Someone or Something Greater was the only scale with no association with the Death Anxiety than the other sub-scales of QLQ-SWB32 in the correlation analysis. The others were all negatively related to death anxiety. When we explored the adjusted relationship between death anxiety, sociodemographic characters, and the QLQ-SWB32 scale scores, we found that death anxiety and demographic variables included as independent variables in the final model associated with the QLQ-SWB32 scale scores. The same result was also found by Nasrin et al*.* [41] among women with cancer indicated an inverse relationship between death anxiety and spiritual well-being. Nevertheless, previous studies have some inconsistent and contradictory findings on this correlation. A survey among Filipino older adults[42] stated no relationship between spiritual well-being and death anxiety. Another study among Christian older adults also reported the absence of a relationship between spiritual well-being and death anxiety[43]. The above contradictory and uncertain findings may be due to the complex mechanism supporting this relationship. It is well documented that the strength or even the direction of the relationship between death anxiety and spiritual well-being may depend mainly on the specific context and sociocultural characteristics of the sample studied[44]. As the conditional studies of this relationship are scarce, little is known about how and under what conditions death anxiety may significantly affect spiritual well-being [44]. Some scholars believe that spiritual well-being brings hope to dying individuals and helps them find meaning in life[45]. Also, individuals with higher spiritual well-being are more likely to accept death as a natural process in life. They can be aware of the inevitability of death and accept it, rather than experiencing excessive anxiety about it[44]. From a terror management theory (TMT) perspective, both religious and supernatural beliefs function as a defense against death anxiety[46, 47]. Religious beliefs can alleviate death anxiety by promising an afterlife and literal immortality[47]. At the same time, spirituality provides a broader framework for personal meaning-making that helps people access symbolic immortality by linking self-worth to transcendence[48, 49].

As for social-demographic characteristics, we found that compared with unmarried/divorced patients, married patients scored higher on the Relationship with Others scale. And those who were living with a partner also scored higher on the Relationship with Others scale. The results were similar to Lazenby's research, which found that spiritual well-being is associated with marital status[50]. It may be related to the enhanced social and family relationship of married or living with a partner and the feeling of more family support.

This study has some limitations. The cross-sectional nature of the study resulted in no causal relationship between the variables included. Most of our subjects had a disease duration of one year or below, and we failed to reach more terminal patients who may have had more thinking about spirituality. So, our findings may not necessarily be generalized to such patients. Besides, previous literature in the western sample showed that females had higher death anxiety than males [44]. Thus, this relationship between death anxiety and spiritual well-being may look different in male patients with cancer or a mixed sample of men and women. Furthermore, the independent variables had a low interpretation of the dependent variables in our multiple linear regression analysis, indicating that many other relevant demographics and clinical variables that affected SWB were not included. For example, a study among Chinese older adults with a disability had found that social support and depression directly affected spiritual well-being, and functional ability indirectly affected spiritual well-being [51]. A study of 436 college students who had survived traumatic experiences suggested that spiritual well-being was an important post-traumatic outcome warranting future research and clinical attention [52].

## Clinical Implications

This study was conducted in the context of no development of formal religion. However, we found that the spiritual well-being of patients in our country was no worse than in other countries with religious beliefs. Spiritual well-being is often associated with religious faith in some studies [53, 54], but it shouldn't be confused with religion. Spirituality is a broader concept, and everyone can experience spirituality, regardless of religious faith[55]. Therefore, clinical staff should pay attention to the spiritual health of cancer patients, and spiritual care should be regarded as an essential element in cancer care. Since we have found a correlation between spiritual well-being and death anxiety, and patients with lower death anxiety have a higher level of spiritual well-being, we could improve spiritual health by reducing their death anxiety. What's more, evidence had proven that some spiritual therapy interventions such as Managing Cancer and Living Meaningfully (CALM)[56], which are important in the spiritual process, can also effectively reduce death anxiety. It is reported that patients with moderate levels of death anxiety could benefit most from CALM therapy because it reduced such distress and improvement of spiritual well-being and attachment security[57]. Therefore, future studies are needed to clarify how and under what conditions death anxiety may impact spiritual well-being and verify the effects of these spiritual intervention therapies for non-religious cancer patients. Besides, future studies could explore how cancer patients without religious beliefs develop spirituality. And to explore what factors influence spiritual well-being, such as meaning and purpose in life, social support, post-traumatic outcome, and other psychological and cancer-related factors, develop more appropriate intervention therapies.

## Conclusion

In conclusion, our study found that the spiritual well-being of patients with gynecological cancer in China was no worse than in other countries with religious beliefs. Clinical staff should pay attention to the spiritual health of cancer patients, and spiritual care should be regarded as an essential element in cancer care. Relationship with Someone or Something Greater was the only scale not associated with death anxiety. Overall, patients with lower death anxiety have a higher level of spiritual well-being. Future studies are needed to clarify how and under what conditions death anxiety may impact spiritual well-being.

## Data Availability

The datasets used and analyzed during the current study are available from the corresponding author on reasonable request.
